# Depictions of Insomniacs' Behaviors and Thoughts in Music Lyrics

**DOI:** 10.1155/2013/106492

**Published:** 2013-01-14

**Authors:** Constance H. Fung, Stella Jouldjian, Lara Kierlin

**Affiliations:** ^1^Geriatric Research Education and Clinical Center (GRECC), Veterans Administration Greater Los Angeles Healthcare System, North Hills, CA 91343, USA; ^2^David Geffen School of Medicine, University of California, Los Angeles, CA 90095, USA; ^3^Oregon Sleep Associates, Portland, OR 97210, USA

## Abstract

*Study Objectives.* Studies have found that depictions of unhealthy behaviors (e.g., illicit substance use, violence) are common in popular music lyrics; however, we are unaware of any studies that have specifically analyzed the content of music lyrics for unhealthy sleep-related behaviors. We sought to determine whether behaviors known to perpetuate insomnia symptoms are commonly depicted in the lyrics of popular music. *Methods.* We searched three online lyrics sites for lyrics with the word “insomnia” in the title and performed content analysis of each of the lyrics. Lyrics were analyzed for the presence/absence of the following perpetuating factors: extending sleep opportunity, using counter fatigue measures, self-medicating, and engaging in rituals or anti-stimulus control behaviors. *Results.* We analyzed 83 music lyrics. 47% described one or more perpetuating factor. 30% described individual(s) engaging in rituals or antistimulus control strategies, 24% described self-medicating, 7% described engaging in counter fatigue measures, and 2% described extending sleep opportunity (e.g., napping during daytime). *Conclusion.* Maladaptive strategies known to perpetuate insomnia symptoms are common in popular music. Our results suggest that listeners of these sleep-related songs are frequently exposed to lyrics that depict maladaptive coping mechanisms. Additional studies are needed to examine the direct effects of exposing individuals to music lyrics with this content.

## 1. Introduction

Insomnia is a prevalent condition among adolescents and adults, with an estimated prevalence of 22.1% when using DSM-IV-TR criteria [1]. Insomnia frequently presents in the context of comorbid conditions, ranging from substance use disorders to medical or psychiatric disorders, or even other sleep disorders such as obstructive sleep apnea. Less commonly, insomnia may be seen as a primary disorder, with the sleep complaint existing outside of other discernible medical or psychiatric cause [2]. In Spielman's 3-P model of insomnia, predisposing (biological and psychological inputs), precipitating (acute stressors), and perpetuating factors (behaviors such as napping that maintain or exacerbate sleep difficulties) contribute to individual sleep disturbance to varying degrees [3]. In particular, perpetuating factors can be seen as modifiable behaviors that may serve as intervention points in insomnia treatment and public health awareness campaigns [4–6]. 

As described by Perlis et al., the four major types of perpetuating factors include (1) extending sleep opportunity (e.g., napping), (2) using counter fatigue measures (e.g., increasing coffee consumption), (3) self-medicating (e.g., drinking alcohol to promote sleep), and (4) using strategies that reduce stimulus control (e.g., reading in bed) or result in anticipatory anxiety if the strategy becomes unavailable (e.g., drinking special teas) [7]. These perpetuating factors contrast with recommended strategies for coping with insomnia, such as keeping a consistent wake up time and getting out of bed when unable to fall asleep. Cognitive behavioral therapy for insomnia, which is the most effective treatment for chronic insomnia, addresses these perpetuating factors through sleep restriction, stimulus control, sleep hygiene, and cognitive restructuring [4]. From a public health perspective, chronic insomnia could potentially be prevented if individuals avoided maladaptive compensatory behaviors when faced with factors that precipitate insomnia.

Studies of other health conditions suggest that the content of popular media may be of interest to clinicians, researchers, and public health programs, because popular media can influence the behaviors of individuals [8, 9]. For example, a systematic review found that exposure to alcohol advertising or alcohol promotional activity in print or broadcast media was associated with subsequent alcohol consumption in young people [8], and a recent study found an association between exposure to cannabis in popular music and early cannabis use among urban American adolescents [9]. Popular media could also affect the way individuals approach their own insomnia symptoms [10]. A study of British print media found that insomnia is couched in terms of stress and anxiety, and that treatments discussed in the media ranged from sleep hygiene to cognitive behavioral therapy to self-help remedies [10]. We are not aware of any published studies of popular music that have assessed the frequency with which perpetuating factors are conveyed.

The goal of this study was to assess the prevalence of the four types of perpetuating factors (extending sleep opportunity, using counter fatigue measures, self-medicating, and engaging in rituals or antistimulus control behaviors) found in the lyrics of popular music.

## 2. Materials and Methods

We searched three online music lyrics sites (http://www.lyrics.com/, http://www.metrolyrics.com/, and http://www.lyricsmode.com/) for lyrics with the word “insomnia” in the title (search completed in August 2012). We selected these licensed and authorized sites, because they pay royalty fees to songwriters and publishers based upon legal agreements between parties involved, unlike other sites that do not have the endorsement of those owning the music being referenced. We downloaded the music lyrics of all songs that appeared in the search results (*N* = 119). These three online sites did provide some lyrics in languages other than English. These were included in the sample, and were translated into English using Google translator. Non-English language lyrics that could not be translated using Google Translate (http://www.translate.google.com/) were attempted to be translated using Microsoft Translator (http://www.bing.com/translator/).

Two trained coders (CHF and LK) reviewed the content of all of the music lyrics downloaded. The ratings of the two coders were compared for each set of music lyrics, and differences were discussed. Residual differences in ratings were resolved completely with the input of a third coder (SJ), who independently reviewed the music lyrics blinded to the initial ratings of the first two coders. During their initial assessment, the coders independently assessed whether the lyrics were interpretable (e.g., sufficient amount of translation from non-English language; *N* = 101) and contained unique lyrics (some lyrics were the same for two different artists; *N* = 112). Music lyrics that were deemed interpretable and that contained unique lyrics were further assessed to determine if the music lyrics provided content pertaining to insomnia. A total of 83 music lyrics were rated as having insomnia content and underwent further evaluation for the presence of maladaptive strategies for coping with insomnia [7] as described below. 

Our research project was approved by VA Greater Los Angeles Healthcare Institution Research and Development.

### 2.1. Measures

1. Extending sleep opportunity (e.g., going to bed early, waking up later, or napping). These strategies may deprime the sleep homeostat (reduce the propensity for sleep) and may result in circadian dysregulation.
2. Engaging in counter fatigue measures (e.g., using stimulants or decreasing physical activity). Ingesting stimulants may increase arousal at inappropriate times, and decreasing physical activity may deprime the sleep homeostat, which could lead to conditioned arousal over time if the individual also spends more time resting in bed.
3. Engaging in rituals or antistimulus control strategies (e.g., engaging in behaviors in the bedroom, sleeping outside the bedroom, using special herbs, teas, etc., to promote sleep and avoiding behaviors thought to inhibit sleep). These strategies may lead to a lack of stimulus control or may lead to anticipatory anxiety if the ritual becomes unavailable.
4. Self-medicating: sedation with nonprescription substances (e.g., increase alcohol intake before bedtime, use of marijuana, use of over-the-counter antihistamines, use of melatonin as a hypnotic, or use of prescription medications not approved for hypnotic use such as opioids or certain benzodiazepines). These substances may disrupt the sleep stages, lead to psychological dependence, decrease sleep-related self-efficacy, cause rebound insomnia, or have the potential to shift circadian phase in the case of melatonin.

Frequency counts (presence/absence of each strategy type) were summarized using Microsoft Office Excel 2007.

## 3. Results

Table 1 provides a list of the music lyrics included in the analysis.  Table 2 provides the frequency of perpetuating factors identified through content analysis. Overall, analysis of the lyrics containing insomnia content revealed that 47% described one or more perpetuating factor, and 14% described two or more perpetuating factors. The most frequent type of perpetuating factor described an individual engaging in rituals or other maladaptive strategies. The following examples depict antistimulus control behaviors:

*“I lie there staring at the dark ceiling and wait… My neurotic brain races for hours about everything possible.” *

*“Hypnotized by the strips on my TV.” *

*“I sit alone and I watch the clock. I breathe in on the tick and out on the tock.”*

The second most frequent perpetuating factor described the use of substances for promoting sleep. The following three examples depict the use of substances for promoting sleep:

*“Poured a bottle off NyQuil in my veins.” *

**“Insomnia's lullaby, drinking wine and counting down the time.”**

* “I only smoke weed when I need to.”*

A few music lyrics described an individual engaging in counter fatigue measures. The following is an example: “But now I keep myself pepped.” Descriptions of an individual's attempt to extend sleep opportunity such as napping during the daytime were the least frequent type of perpetuating factor. The following are two examples of extending sleep opportunity: “I might sleep all day” and “Gotta try sleep through to Saturday.” 

## 4. Discussion

To our knowledge, this is the first study to evaluate the content of popular music for insomnia coping strategies. Our study found that maladaptive strategies for coping with insomnia symptoms are common in popular music, with close to half of the music lyrics referencing at least one perpetuating factor. Behaviors that adversely affect stimulus control (e.g., tossing and turning in bed) and the use of substances such as alcohol, marijuana, or opioids were the most commonly described behaviors. Our results suggest that listeners of these sleep-related songs are frequently exposed to unhealthy coping mechanisms for addressing insomnia symptoms. 

The high prevalence of maladaptive strategies contained in popular music lyrics is colored by the dramatic nature of the medium, but it is also a reflection of the ways in which the public, in general, copes with sleep disturbances. A 2005 National Sleep Foundation survey found that 14% of Americans surveyed used sleep aids at least a few nights per week, 13% used alcohol within one hour of going to bed at least a few nights per week, and 35% took naps at least two times per week [11]. One market research study estimated that the worldwide market for sleeping pills may expand to $9.0 billion by 2015 [12]. Research has demonstrated that insomniacs tend to have increased belief in the negative sequelae of their sleep difficulty, which may itself be a perpetuating factor, and presentation of insomnia as highly dramatic and worthy of distress (e.g., “I'm hangin on by a thread to my sanity”) may itself increase anxiety relating to sleep and worsen insomnia [13].

Our findings are important because popular music reaches a wide audience and can impact the sleep of listeners. American adolescents and younger children listen to 1.5–2.5 hours per day to music [14]. Many songs have been written to influence sleep (e.g., Johannes Brahms's *Guten Abend, gute Nacht*). A positive relationship between music and initiating sleep has been recognized for many years. In 1928, Frederico Garcia Lorca delivered his lecture, “On Lullabies,” which describes the soothing effects of lullaby lyrics on children's sleep [15]. More recently, two randomized controlled studies that assessed the effects of listening to music at bedtime found that music improves self-reported sleep duration and sleep efficiency [16, 17]. Another study of young adults found that listening to classical music at bedtime improves sleep quality and reduces depressive symptoms as compared with an audiobook or control [18]. Although studies examining the effect of negative lyric content on sleep behaviors are not available, studies of other types of behaviors show an association between music lyrics and behavior. For instance, a strong relationship has been found between violent music lyrics and aggressive behavior [19, 20]. Other studies suggest that exposure to sexual messages and use of substances in music videos might change adolescents' behaviors and attitudes [21]. 

The 2006 Institute of Medicine report, “Sleep Disorders and Sleep Deprivation: An Unmet Public Health Problem” [22], described a need for a multimedia public education and awareness campaign to promote healthy sleep practices. Popular music remains untapped as a vehicle for raising awareness about sleep deprivation from insomnia and the behaviors that perpetuate insomnia symptoms. We already know that music lyrics can also have a positive impact on behavior. A study by Greitemeyer found that listening to songs with prosocial, relative to neutral, lyrics increased helping behavior. In theory, music lyrics could promote healthy sleep habits and encourage use of recommended strategies for dealing with events that trigger insomnia. Highlighting healthy sleep habits in songs that have been released and/or offering incentives for artists to write prosleep lyrics are two ways public health campaigns could use popular music to promote healthy sleep. Lyrics by Milk Inc, for example, describe an individual who has gotten out of bed due to insomnia symptoms, which is a behavior that promotes stimulus control: “At the count of three I pull back the duvet. Make my way to the refrigerator…But there's no relief. I'm wide awake in my kitchen.” The following lyrics by Vivacity provide an example of an individual who challenges his/her beliefs about poor sleep, which may reduce the anxiety and arousal that can perpetuate insomnia (cognitive restructuring): “Go on just leave me here, cause this night is going to hell. Well it's just one more night, We'll make this time turn out well.” Other forms of media such as television have successfully incorporated positive health messages into programming. For example, The Henry J. Kaiser Family Foundation has partnered with MTV, Univision, and Fox Networks Group to reduce sexually transmitted diseases, substance abuse, and obesity [23]. 

Our study has several limitations. First, because we only searched for music lyrics with the word “insomnia” in the title, we did not capture the lyrics focusing on insomnia whose song titles did not include “insomnia.” Our search strategy, however, identified music lyrics that would have a high likelihood of containing content about insomnia. Second, although we captured some non-English lyrics, which we attempted to translate using publically available translation tools, we found that we were not able to translate all of the non-English lyrics and had limited access to other means of translation. Similarly, we were not able to use other languages for our search term because our research team did not have the capability to translate other languages. It should also be noted that since the sample included few non-English language lyrics, generalizing the findings to all music and/or English language media should be done with caution. 

## 5. Conclusion

Many popular music lyrics that focus on insomnia depict unhealthy coping strategies for addressing sleep disturbance. Future studies should examine the direct effect of exposing individuals to music lyrics with a range of content (perpetuating factors versus recommended strategies for coping with insomnia). Additional studies of other forms of popular media, such as music videos, television, and social media exchanges, are needed to obtain better understanding of the types of messages that are conveyed in other forms of media and their influence on health behavior. Public health campaigns could consider using popular music to promote healthy sleep habits. Studying media's depictions of insomnia symptoms not only may inform our understanding of its impact on individual behavior and perceptions, but also could result in more targeted public health campaigns.

## Figures and Tables

**Table 1:** Song titles and artist names of music lyrics analyzed (*N* = 83).

Artist Name	Song Title
Abgott	Shining Insomniac
Abysmalia	Whispering Insomnia
A C T	Insomniac
Annihilator	Insomniac
Anssi Kela	Insomnia
Anxiety of Influence	Insomniac
Bif Naked	Insomnia
Big K R I T	Insomnia
Billy Pilgrim	Insomniac
Bleeding Through	Insomniac
Boiler Room	Insomnia
Carpathian	Insomnia
Charlie	Insomnia's Lullaby
Counting Crows	Up All Night (Frankie Miller Goes To Hollywood)— The Song Formely Known As Insomnia
Craig David	Insomnia
Crüxshadows	Insomnia (A Ghost Story)
Dark Age	Insomnia
Deinonychus	Nightfall Guides Insomnia to be an everlasting mental torture, With This Being The Concequence
Dirty Heads	Insomnia
Dr. Sin	Insomnia
Duna Hill	Jack Insomniac
Echobelly	Insomniac
Electric President	Insomnia
Empyrios	Insomnia
Enter Shikari	Insomnia
Entwine	Insomniac
Faithless	Insomnia
Feeder	Insomnia
Finch	Insomniatic Meat
Government Issue	Insomniac
Grave Flowers	Insomnia
Gutter Demons	Insomnia
Haken	Insomnia
Her Six Daughters	Insomnia
Imago Mortis	Insomnia
Isole	Insomnia
Jester's Funeral	Insomnia l
Jill Scott	Insomnia
Katatonia	Tomb of Insomnia
Kylesa	Insomnia for Months
Ligion	Insomniacs Dreams
Live	Insomnia and the hole in the universe
Luka Belani	Love Insomnia
Lumine Criptica	Insomnia
Lunachicks	Insomnia
Make Do And Mend	Insomniac Jams
Marillion	Insomnia
Megadeth	Insomnia
Minus	Insomniac
Music Emporium	Insomnia
Mustard Plug	Insomnia
Nephenzy Chaos Order	Insomnia
No More Heroes	Insomnia
Noe Venable	My insomnia
Pensive	Insomnia
Periphery	Insomnia
Raventhrone	Malicia The 3rd (Empress Of Insomnia)
Rentals	Insomnia
RZA	Insomnia
Salad	Insomnia
Sea Of Desperation	Insomnia
Shawn Desman	Insomniac
Shining Star	Insomnia
Silverchair	Insomnia
Slaine	Insomnia
Slowmotion Apocalypse	The Insomniac
Sum 41	Hyper-Insomnia
The Glass Child	Insomnia
The Veronicas	Insomnia
Tyler Hilton	Insomnia
Vader	Insomnia
Various Artist	Insomnia
Vivacity	Insomnia
War From A Harlots Mouth	Insomnia
Wheesung and Craig David	Insomnia
White Willow	Insomnia
Wintersleep	Insomnia
Wishbone Ash	Insomnia
Wynter Gordon	Insomnia
Wyrd	Ominous Insomnia
Yellow 5	Heart Break Insomnia

**Table 2:** Characteristics of perpetuating factors identified in music lyrics (*N* = 83).

Number of perpetuating factor(s) per music lyric	*N* (%)
None	44 (53)
One	27 (32)
Two	8 (10)
Three	4 (5)
Four	0 (0)

Type of perpetuating factor	*N* (%)

Engaging in rituals or anti-stimulus control strategies	25 (30)
Self-medicating	20 (24)
Engaging in counter fatigue measures	6 (7)
Extending sleep opportunity	4 (2)
